# Response Analysis of PLC Optical Splitters Under Force Cyclic Loading

**DOI:** 10.3390/mi16040449

**Published:** 2025-04-10

**Authors:** Lianqiong Jiang, Yu Zheng, Ke Zeng, Xin Tang

**Affiliations:** 1 State Key Laboratory of Precision Manufacturing for Extreme Service Performance, College of Mechanical and Electrical Engineering, Central South University, Changsha 410083, China; zhengyu@csu.edu.cn (Y.Z.); 223701029@csu.edu.cn (K.Z.); tangxin.cmee@csu.edu.cn (X.T.); 2 College of Mechanical and Electrical Engineering, Wuyi University, Wuyishan 354300, China

**Keywords:** PLC optical splitters, force cyclic loading, adhesively bonded joint, insertion loss

## Abstract

In order to better understand the damage phenomenon and failure mechanism of planar lightwave circuit (PLC) optical splitters under force cycling, this paper established an online test experimental platform to study their optical and mechanical performance response under the action of force cycling. The research results show that under the action of force cyclic loading, the weakest area in the PLC optical splitter is the eight-channels output fiber array–PLC chip adhesively bonded joint; the moments of force cycle loading and unloading cause the insertion loss (*IL*) of the PLC optical splitter to fluctuate suddenly, especially at the moment of unloading. In addition, the research results show that under the action of force cyclic loading, the local deformation and damage behavior of the weak area can be reflected by the optical performance parameter indicators monitored in real time. This study helps to identify the location of weak areas of PLC optical splitters and understand their response behavior under force cyclic loads, which can provide a useful reference for subsequent measures to improve their long-term reliability.

## 1. Introduction

In optical communication networks, planar lightwave circuit (PLC) optical splitters can realize the control of optical signal transmission direction, the distribution of optical signal power and the coupling control between devices, etc., which are indispensable key devices in fiber-to-the-home (FTTH) [1,2,3]. Along with the surge in demand for PLC optical splitters, the market is demanding higher reliability to minimize the maintenance and operation costs of optical networks [4,5]. According to the principle, optical splitters can be divided into fused biconical taper (FBT) optical splitters and PLC optical splitters [6]. Since the PLC optical splitter adopts optical integration technology and integrates the splitting function on the chip, it has many fewer splicing points than the FBT optical splitter. The corresponding quality hazards are greatly reduced and the reliability is better. In addition, the PLC optical splitter has the characteristics of wavelength insensitivity, small size, high reliability, good light splitting uniformity, and wide operating wavelength range. Therefore, as the price of PLC optical splitter continues to decline, it has basically replaced FBT optical splitters. PLC optical splitter will face complex load conditions during service, such as cyclic or pulse loads due to repeated mechanical effects and fluctuating temperature environments. This leads to the degradation of the optical performance of the PLC optical splitter and the failure of its structurally weak areas [7,8]. Therefore, the fatigue behavior of PLC optical splitter is a problem that cannot be ignored.

The research on PLC optical splitters so far can be roughly divided into two categories. One category is to conduct environmental and mechanical reliability testing of existing products according to relevant standards (such as Telcordia GR-1209/1221-CORE) [9,10] to determine whether it has long-term stable optical performance and analyze the possible reasons for its failure [11,12,13]. The other focuses on the structural optimization design and functional implementation of PLC optical splitters [14,15,16,17,18]. During long-term service, PLC optical splitters may be subjected to cyclic loads, such as force cycles, temperature cycles, and vibrations [5,19]. These external factors can cause stress concentration or damage to their internal structures, thus affecting the stability of their optical performance. As cyclic loads are continuously applied, the optical performance of PLC optical splitters may gradually deteriorate, thereby affecting the reliability and efficiency of the entire optical communication system. However, there is currently almost no research on the optical performance response of PLC optical splitters under mechanical cyclic loads. This research gap limits our comprehensive understanding of their performance.

For PLC optical splitters, in many cases, slight changes in their structure may cause the rapid deterioration of their optical performance or complete failure [7,20]. During the cyclic loading process, the failure process of the PLC optical splitter is actually the gradual accumulation of material deformation or damage. Therefore, in situ monitoring of optical properties may prove suitable for obtaining information on structural damage evolution. This paper builds an online experimental test platform to study the optical and mechanical performance response of the 1 × 8 PLC optical splitter under force cyclic loading. This research not only helps to understand the failure mechanism of PLC optical splitters, but also provides guidance for improving design and material selection, thereby improving their reliability.

## 2. Experimental Work 

### 2.1. Structure of the PLC Optical Splitter

**Figure 1:**
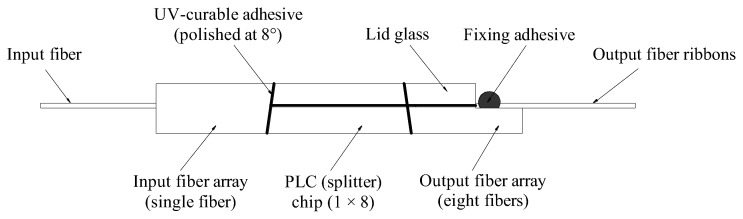
Structural diagram of the internal core structure of a 1 × 8 PLC optical splitter [23].

This study selected the same batch of 1 × 8 PLC optical splitters produced by Company A (Guangzhou, China) as the research object. Figure 1 illustrates the typical internal core structure of a 1 × 8 PLC optical splitter after the stainless steel housing is removed. The structure primarily consists of three components: a 1-channel input fiber array, an 8-channel output fiber array, and a PLC (splitter) chip. These components are precisely aligned along the optical axis and bonded using UV-curable adhesive to achieve optical and mechanical integration [13,15]. The end faces of the fiber arrays and the PLC chip are polished at an 8° angle to enhance return loss and ensure stable optical power output [21,22]. As shown in Figure 1, both the fiber array and the PLC chip structure, respectively, include a lid glass with a thickness of ~1 mm, which serves to fix the fiber and protect the PLC chip. The fiber array has a rectangular cross-section with dimensions of W × H (width × height) = 2.5 × 2.5 mm^2^, and the cross-sectional dimensions of the PLC chip are approximately equal to it.

Since PLC chips and fiber arrays of silicon-based/quartz glass materials are chemically and physically stable, the bonding performance between the fiber array and the PLC chip has a significant impact on the reliability of the optical waveguide splitter. In other words, the deterioration of the optical properties of PLC optical splitter is mainly caused by the deformation, delamination, or debonding of the adhesive [5,23]. The UV-curable adhesive used for interconnection between the fiber array and the PLC chip in this study is an acrylate adhesive with an elastic modulus of 20 MPa, and a curing condition of 10 mW/cm^2^, a curing time of 5 min, which complies with the Telecodia standard (high temperature and high humidity) and has good flexibility.

### 2.2. Test Method

**Figure 2:**
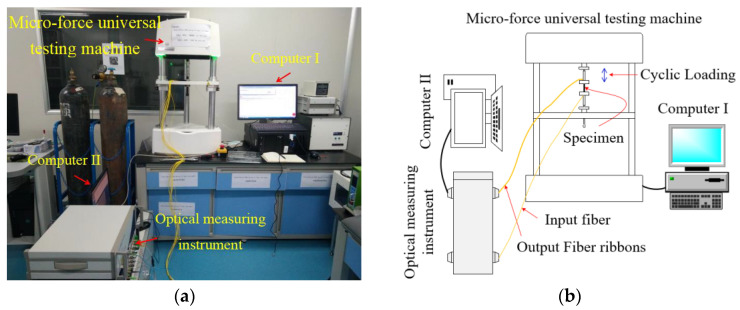
Schematics of the experimental setup: (**a**) photo of the experimental setup; (**b**) schematic diagram of an experimental system.

In order to simultaneously test the mechanical performance and optical performance response of the PLC optical splitter under force cycling, an online experimental test platform was built, as shown in Figure 2. The online experimental test platform mainly includes a micro-force universal testing machine (ElectroForce^®^ 3200 Series III developed by TA instrument, New Castle, DE, USA) for force cycle testing and an optical measuring instrument (Viavi MAP-200 Multi-Application Platform, San Jose, CA, USA) for in situ monitoring of the optical performance of optical devices, as well as the corresponding Computer I and Computer II. The control of the micro-force universal testing machine is provided by the WinTest^®^ digital control system (TA Instruments, New Castle, DE, USA) installed in Computer I, which provides waveform generation, data acquisition and instrument control. This system is suitable for various static and dynamic testing experiments. The WinTest^®^ digital control system provides real-time window for observing load and displacement waveforms and data, and useful data can be collected and saved. The optical measuring instrument (Viavi MAP-200 Multi-Application Platform) is an ideal instrument for measuring optical properties such as insertion loss (*IL*), polarization-dependent loss (*PDL*), and return loss (*RL*). As shown in Figure 2, the input fiber and output fiber ribbons of the sample are connected to the corresponding ports of the optical measuring instrument to form a closed loop. Computer II adjusts and controls the optical measurement instrument by operating the corresponding software, enabling in situ monitoring of the optical performance at each output port of the sample while continuously recording and saving the corresponding data. In the process of force cycle experiments on PLC optical splitters, the testing activities of the online test platform mainly include the following two aspects: (1) mechanical performance testing, that is, the mechanical performance response of the specimen under force cycling; (2) optical performance testing, that is, in situ monitoring of the optical performance parameter index *IL* of the specimen during the force cycle experiment.

**Figure 3:**
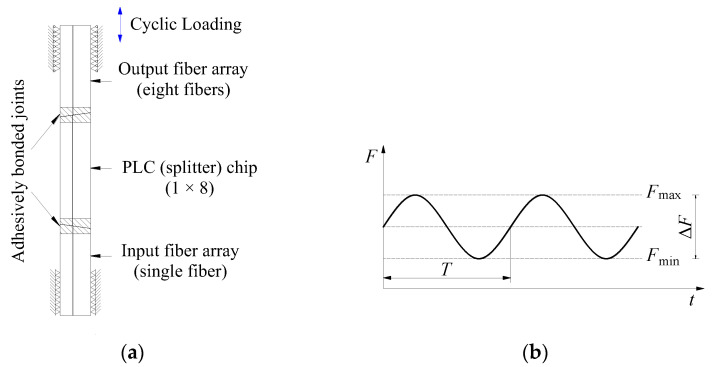
Schematic diagram of force cycle experiment: (**a**) loading and restraint; (**b**) loading waveform.

The loading and constraint diagram of the internal core structure of the PLC optical splitter on the micro-force universal testing machine is shown in Figure 3a. When the PLC optical splitter is placed on a micro-force universal testing machine for uniaxial force cycle experiments, in order to avoid the 1-channel input fiber being damaged or broken due to bending or introducing additional optical loss, the 8-channels output fiber array is placed above, the 1-channel input fiber array is placed below, as shown in Figure 3a. When the micro-force universal testing machine is working, the lower clamp is fixed and the upper clamp drives the specimen to perform uniaxial force cyclic motion. The force cyclic loading waveform is shown in Figure 3b.

The stress ratio *R* = 0 was set for this force cycle experiment (where *R* = *F*_min_/*F*_max_, *F*_min_ and *F*_max_ are, respectively, the minimum and maximum load values applied in the force cycle). The load value causing fatigue failure is often smaller than the safe load estimated based on static fracture analysis [24,25]. Therefore, based on the average failure load obtained by the authors in the previous uniaxial tensile fracture test on the PLC optical splitter (see reference [23] for details), this paper takes *F*_max_ values smaller than this for force cycling tests. The parameters of force cycle testing are summarized in Table 1. All tests were conducted in the same laboratory environment (*T* = 21 ± 2 °C, RH = 40 ± 5%) to minimize the effects of ambient temperature changes.

The force cycle experiment adopts the uniaxial pull–pull cycle loading method under the force control mode. As shown in Table 1, the loading frequency and stress ratio are kept unchanged during the experiment, and the load waveform adopts a sine wave with constant amplitude, where the loading frequency is set as 10 Hz, the stress ratio is set to *R* = 0. According to the uniaxial tensile test results, the maximum load value *F*_max_ of this force cycle is set as 10 N and 8 N, respectively. *F*_max_ is set to 10 N and 8 N, respectively, for the following two situations: (1) *F*_max_ is set to 10 N to examine the response of the specimen from the initial force cyclic loading until complete fracture; (2) *F*_max_ is set to 8 N to examine the response of the specimen from the initial force cyclic loading to the artificial active unloading of the specimen before it breaks. At least five specimens were tested under each condition to verify the reproducibility of the results. Before the experiment starts, the displacement and load data in the data acquisition system are cleared, respectively. After the experiment starts, the displacement and load data are collected by the “Disp” and “Load” channels in the data acquisition system, respectively. Both sets of data are time domain signals, and each moment corresponds to a set of test points.

In actual service, PLC optical splitters usually suffer from two types of failures, namely optical performance failure and mechanical performance failure. For a PLC optical splitter, if its optical performance changes significantly during use so that it exceeds the given standard (for example, the absolute value of Δ*IL* > 0.5), it is considered an optical performance failure; if its structure is broken, it is considered a mechanical interconnection failure. In the force cycle experiment of the PLC optical splitter, a 1 × 8 PLC optical splitter and an optical measuring instrument formed a loop to monitor the changes in *IL* of the eight output ports in real time during the experiment. Based on the recorded *IL* data, the impact of force cycling on the optical performance of the PLC optical splitter and its damage status under force cycling were evaluated. In this force cycle experiment, when the specimen breaks, the number of cycles at this time is recorded. Regarding the fracture of the specimen, in addition to observing the fracture state of the specimen with the naked eye, there are usually two more accurate real-time indicators: (1) The original regular cyclic waveform in the observation window provided by the WinTest^®^ digital control system shows obvious distortion; (2) In the observation window matched with the optical measuring instrument, the *IL* values of the eight output ports of the specimen changed drastically. When the above two situations occur, the specimen structure can be considered to be broken.

## 3. Results and Discussion

Through observations and the experimental results statistics, it was found that the fracture locations of the specimens almost all occurred at the eight-channels fiber array–PLC chip adhesively bonded joint, which means that this is the weak point of the PLC optical splitter. For the fractured specimens, an optical microscope was used to observe the optical microscopic morphology of the failed surface, and it was found that they were all mixed failure modes, that is, there were both interface failures and cohesive failures.

Here, we focus on analyzing the following two situations: (1) representative results obtained from the initial force cyclic loading until fracture of the specimen (*R* = 0, *F*_max_ = 10 N); (2) representative results obtained from the initial force cyclic loading to active unloading of the specimen (the specimen has not yet broken) (*R* = 0, *F*_max_ = 8 N). Note that although only selected results are presented here, they are representative of similar behavior exhibited by most other specimens.

### 3.1. The Results of Cyclic Loading Until the Specimen Breaks

#### 3.1.1. Mechanical Test Results

**Figure 4:**
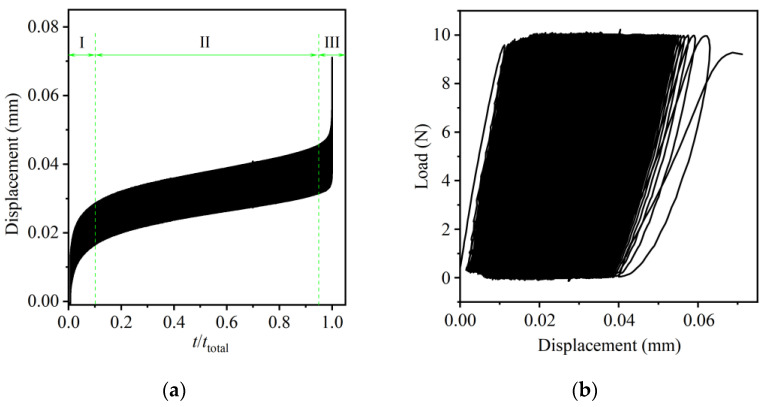
Typical results under constant amplitude force cyclic loading: (**a**) relationship between displacement and normalized time; (**b**) load–displacement curve.

**Figure 5:**
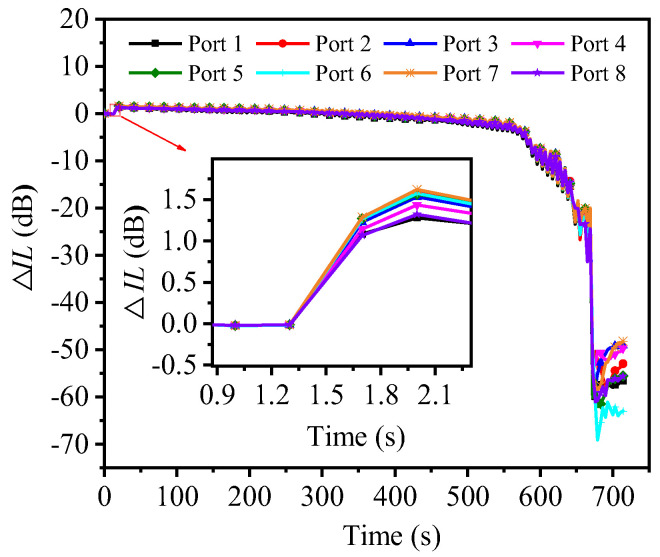
Typical Δ*IL*-time curves at 1550 nm for each output port of a 1 × 8 PLC optical splitter from the beginning of force cyclic loading until fracture (due to the large number of data points, only a portion of the data points are selected at intervals and shown on the curves with the corresponding identifiers for the sake of distinguishing the individual curves).

The experimental results of a 1 × 8 PLC optical splitter specimen from the initial force cyclic loading until fracture were analyzed in detail. Figure 4a shows a typical displacement response versus normalized time, and Figure 4b shows the corresponding load–displacement curve (hysteresis curve) plot. The relationship between displacement and normalized time (Figure 4a) is the change curve of the axial displacement of the specimen, with time recorded by the data acquisition system of the micro-force universal testing machine. Since the elastic modulus of the optical fiber array and PLC chip materials is much larger than that of the UV-curable adhesive material, the deformation under small loads is negligible, and the acrylate-type UV-curable adhesive is ductile [26]. Therefore, the displacement of the specimen in the uniaxial tensile direction can be considered to be mainly caused by the deformation, damage and debonding of the UV-curable adhesive layer. However, part of it may also be caused by the adaptability of the equipment and the clamping slight slippage of the held specimen during the force cycle. The load–displacement curve shown in Figure 4b intuitively shows the relationship between the stress and displacement of the specimen. Combining Figure 4 and Figure 5, it can be reflected that as the number of force cycles increases, the deformation and damage of the UV-curable adhesive layer continue to accumulate, resulting in the deterioration of the optical properties of the specimen. When the damage accumulates to a certain extent, the UV-curable adhesive layer rapidly expanded and caused the adhesively bonded fiber array–PLC chip joint to completely break.

The curve in Figure 4a can be divided into three obvious stages: the initial stage (I), the stable stage (II), and the instability stage (III). This curve is very similar to the conventional creep curve [27]. Observing the curve in Figure 4b, we find that the corresponding curve also presents three different stages, from the initial unstable stage, to the intermediate stable stage, to the final unstable stage. The first two stages can be considered as the fatigue crack nucleation and fatigue microcrack expansion stages, accounting for about 95% of the fatigue life. In the last stage, as fatigue damage accumulates, the UV-cured adhesive layer gradually loses its load-bearing capacity and breaks rapidly. In addition, it is noted that as the force cycle proceeds, the overall slope of the curve in Figure 4b gradually becomes smaller, indicating that the rigidity of the specimen structure decreases and the energy loss increases.

#### 3.1.2. Optical Performance Response

Generally, the *IL* value of a PLC optical splitter is stable. If there is a significant change that cannot be recovered, it means that the PLC optical splitter is no longer working properly and is considered to have failed. The main reason for the obvious changes in the *IL* of each output port of the PLC optical splitter under the force cyclic load is that cracks or debonding occur at the optical fiber array–PLC chip adhesively bonded joint, resulting in deviations in the coupling alignment of the optical fiber and the PLC chip, thereby increasing the optical loss. The experimental data obtained from different specimens were processed separately, and it was found that even under the same loading level, the changes in *IL* values of the eight ports of different specimens over time still showed a certain degree of dispersion. However, through the analysis and summary of the experimental phenomena, the experimental results still show some systematic trends, indicating the reliability of the experimental results.

Figure 5 shows the typical Δ*IL*–time curve (wavelength λ = 1550 nm) of each output port obtained through in situ monitoring of the optical measuring instrument during the process of 1 × 8 PLC optical splitter specimen from initial force cyclic loading until fracture. Observing the curves in Figure 5, we find that they have similar change patterns, and they all present three stages, namely, the initial rapid positive growth stage of Δ*IL*, the basic stable change stage in the middle, and the final sharp drop stage. Among them, Δ*IL* is in a basically stable change stage about 90% of the time, which indicates that no large cracks and expansion behaviors occur in the fiber array–PLC chip adhesively bonded joint during this stage. However, the Δ*IL* at this stage fluctuates slightly, which indicates that the structure of the fiber array–PLC chip adhesively bonded joint undergoes slight deformation and continues to accumulate damage. In fact, the UV-curable adhesive layer undergoes reciprocating deformation during cyclic loading, causing deviations in the coupling alignment of the optical path between the optical fiber and the PLC chip, thereby causing small reciprocating fluctuations in Δ*IL*. In addition, this phenomenon may also be related to the elastic–optical effect in optical media. The elastic–optical effect refers to the change in the refractive index of optical materials, such as crystals and glass, under external stress [28,29,30,31]. Previous studies have shown that this change is typically proportional to the applied stress [12,32]. Therefore, during the force cycling test, as the stress on the specimen fluctuates in a nearly periodic manner, the refractive index of the internal medium may also exhibit corresponding quasi-periodic variations. These fluctuations in refractive index can, in turn, affect the optical performance of the device. Based on this, we speculate that the elastic–optical effect may be one of the contributing factors to the small reciprocating fluctuations in Δ*IL* observed during the relatively stable stage. As can be seen from Figure 5, Δ*IL* has a sharp drop stage after the basically stable change stage, which indicates that at this stage, large cracks appear at the fiber array–PLC chip adhesively bonded joint and rapidly expand to complete fracture. In summary, it can be seen that the Δ*IL*–time curve can reflect the process from damage accumulation to sudden fracture in the weak area of the specimen.

It is worth noting that Figure 5 shows that the optical interconnection failure occurred in the first few cycles of the specimen under cyclic loading, while the mechanical interconnection failure occurred after approximately 6000 cycles. This shows that under the action of force cycling, the impact on optical interconnections is greater than the impact on mechanical interconnections. In general, the failure of optical performance precedes the failure of mechanical interconnections. Under the action of cyclic loading, Δ*IL* initially showed a positive growth. The possible reason is that the residual stress and corresponding coupling alignment micro-displacement are inevitably introduced during the clamping process of the specimen. Generally, after the specimen is clamped stably and the data acquisition system of the micro-force universal testing machine is cleared, it is found that its displacement and load values are negative values infinitely close to zero, which means that the specimen is subjected to is the compressive residual stress. Therefore, under the action of tension–tension cyclic loading, the compressive residual stress and the corresponding coupling alignment micro-displacement of the specimen are eliminated in a short time, making the optical performance of the specimen perform better. Subsequently, under the action of force cyclic loading, the Δ*IL*–time curve reflects the accumulation of damage in the weak parts of the specimen. Δ*IL* shows a negative growth trend with the number of cycles until the specimen is completely broken, resulting in a sharp drop in Δ*IL*.

### 3.2. Results from Force Cycle Loading Until Active Unloading

**Figure 6:**
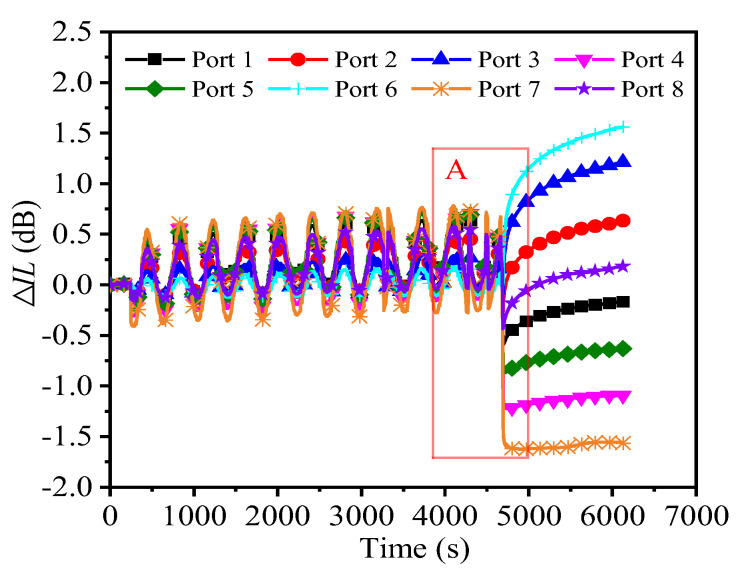
Typical Δ*IL*–time curves at 1550 nm for each output port of a 1 × 8 PLC optical splitter from clamping stabilization, to the start of force cycling, to active unloading (the specimen has not yet broken). (Due to the large number of data points, only a portion of the data points are selected at intervals and shown on the curves with the corresponding identifiers for the sake of distinguishing the individual curves).

During the service of a PLC optical splitter, it is usually not subjected to continuous force cyclic loads until the device breaks, but rather to bear alternating loads within a short period of time. Although mechanical interconnection failure will not occur, it will affect the stability of optical performance. Therefore, this paper conducts a detailed analysis of the *IL* changes in each port during the process from initial loading to active unloading of the specimen (the specimen has not yet broken) to further study the impact of force cycle loading on the optical properties of the specimen. Note that the loading and constraint conditions of the experiment are the same as those described in Section 2.2, but before the specimen breaks, the loading of the specimen is manually stopped (that is, active unloading). Figure 6 shows the typical Δ*IL*–time curve of each output port of the 1 × 8 PLC optical splitter specimen during the process from stable clamping to the beginning of force cycle loading to active unloading (the specimen has not yet broken) (λ = 1550 nm).

As shown in Figure 6, during the entire experiment, the Δ*IL*–time curve showed the following characteristics: (1) the Δ*IL*-time curve of each output port had a similar change pattern before active unloading. However, after unloading, it showed obvious of difference. At the loading and unloading moments, the Δ*IL*–time curve of each output port shows significant changes in a short period of time. Compared with the loading moment, the degree of change at the unloading moment is greater. This may be related to the accumulation of damage in weak areas due to force cycling prior to unloading. (2) After active unloading, due to the sudden loss of the external force cyclic load, the *IL* of each output port shows a jump change almost simultaneously, some of which are positive growth, some are negative growth, and finally, the *IL* of each output port remains at a relatively stable value. This phenomenon shown in the experimental results is similar to the results in the literature [33]. The research in this literature found that the damage caused by the bonded structure during the fatigue test can release the residual stress inside it and make the uneven stress and strain field, resulting in serious changes in the spectral waveform of optical devices. (3) During the constant–amplitude force cyclic loading process, the Δ*IL*–time curve of each output port of the specimen shows a sinusoidal change pattern. As the number of cycles increases, the amplitude of Δ*IL* shows an increasing trend. The occurrence of this phenomenon may be mainly related to the deformation and damage accumulation at the optical fiber array–PLC chip adhesively bonded joint; another possible factor is the elastic–optical effect, that is, the refractive index of the optical medium changes accordingly under the action of alternating stress, thus causing optical performance disturbance. This further indicates that the local deformation and damage behavior of the weak parts of the PLC optical splitter under force cyclic loading can be reflected in its optical performance parameter indicators of its being monitored in situ.

**Figure 7:**
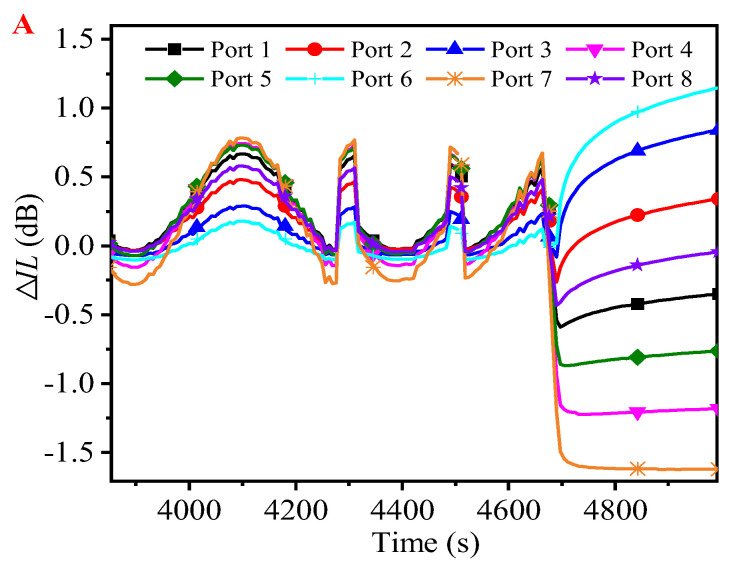
An enlarged view of selection A in the red rectangular frame in Figure 6 (due to the large number of data points, only a portion of the data points are selected at intervals and shown on the curves with the corresponding identifiers for the sake of clearly distinguishing the individual curves).

In order to observe the Δ*IL* of each output port of the specimen in more detail before and after unloading, the rectangular red box area in Figure 6 is enlarged for observation, and the results are shown in Figure 7. It is noted that the waveform before unloading in Figure 7 has obvious distortion, which indicates that the structure of the specimen has suffered a certain degree of mechanical damage before unloading. In addition, by observing Figure 7, it is found that the Δ*IL* value of each output port after unloading has a certain relationship with the change amplitude of Δ*IL* before unloading. The ports (Port 6, Port 2, Port 3, and Port 8 in Figure 7) with relatively small changes in Δ*IL* before unloading have their Δ*IL* restored in the positive direction after unloading; conversely, the ports (Port 1, Port 5, Port 4, and Port 7 in Figure 7) with relatively large changes in Δ*IL* before unloading have their Δ*IL* restored in the negative direction after unloading. The reason for this phenomenon may be that ports with a relatively small change in Δ*IL* before unloading are relatively less affected by the cumulative damage of the UV adhesive layer and the micro-displacement deviation of the coupling alignment. Therefore, when the effect of the force cyclic loading disappears, its optical performance can be restored relatively better.

In summary, it can be seen that the moment of loading and unloading of force cyclic loading causes jump fluctuations in the optical performance of the PLC optical splitter, and the moment of unloading causes greater fluctuations than the moment of loading. In addition, through statistical experimental results, it is found that the change in optical performance with time under the force cyclic load of Δ*F* = 8 N is usually significantly better than that of Δ*F* = 10 N. Therefore, it is reasonable to believe that if corresponding measures are taken to improve the cyclic load and impact resistance of the PLC optical splitter, the long-term stability and reliability of its optical performance during service can be improved.

This paper investigates the performance response characteristics of PLC optical splitters under force cyclic loads by building an online test platform. The aim is to uncover the mechanisms influencing performance and establish a solid foundation for enhancing their long-term reliability and stability. Generally speaking, optimizing the packaging structure and material selection can effectively distribute stress concentration, thereby enhancing the stability of the PLC optical splitter under cyclic loading and delaying the failure process. Therefore, future research should integrate finite element simulation with experimental verification to conduct in-depth studies on structural optimization and material selection for PLC optical splitters, with the aim of proposing specific improvements to enhance their resistance to cyclic force loads and overall reliability.

## 4. Conclusions

This paper analyzes the mechanical performance response and optical performance response of PLC optical splitters under cyclic loading. By analyzing the response behavior of the PLC optical splitter under force cyclic loading, the weak areas in the structure can be identified and its fatigue behavior under cyclic loading can be understood. The conclusions can be summarized as follows:

- The weakest area of the PLC optical splitter under force cyclic load is the eight-channels output fiber array–PLC chip adhesively bonded joint.
- When the PLC optical splitter is subjected to a force cyclic loading with a sinusoidal waveform of constant amplitude, the Δ*IL*–time curve of each output port of the PLC optical splitter also presents a quasi-sinusoidal waveform during the stable loading stage. This further illustrates that the local deformation and damage behavior of the weak area under force cyclic loading can be reflected in its optical performance parameter indicators that are monitored in real time.
- At the moment of loading and unloading of the force cyclic loading, the Δ*IL* of each output port of the PLC optical splitter shows large fluctuations, especially at the moment of unloading.

## Figures and Tables

**Table 1:** Summary of force cycle testing parameters.

Experimental Equipment	Micro-Force Universal Testing Machine, Optical Measuring Instruments, etc.
Specimen	1 × 8 PLC optical splitter
Load type	Constant amplitude sinusoid load level
Load frequency	10 Hz
Stress ratio	*R* = 0
Maximum	*F*_max_ = 10 N or *F*_max_ = 8 N

## Data Availability

The original contributions presented in this study are included in the article. Further inquiries can be directed to the corresponding author.

## Abbreviations

The following abbreviations are used in this manuscript:

PLC	Planar lightwave circuit
IL	Insertion loss
FTTH	Fiber-to-the-home
FBT	Fused biconical taper
PDL	Polarization-dependent loss
RL	Return loss
